# Identification of FXYD6 as the novel biomarker for glioma based on differential expression and DNA methylation

**DOI:** 10.1002/cam4.6752

**Published:** 2023-12-13

**Authors:** Weiliang Hou, Jing Cai, Pei Shen, Shuo Zhang, Siyu Xiao, Pu You, Yusheng Tong, Kaicheng Li, Zengxin Qi, Hao Luo

**Affiliations:** ^1^ Department of Neurosurgery, Huashan Hospital, State Key Laboratory of Medical Neurobiology, MOE Frontiers Center for Brain Science and Institutes of Brain Science Fudan University Shanghai China; ^2^ Department of Oral Surgery, Shanghai Ninth People's Hospital Shanghai Jiao Tong University School of Medicine Shanghai China; ^3^ Shanghai QuietD Biotechnology Co., Ltd. Shanghai China; ^4^ Department of Rehabilitation, Gongan Hospital Hubei University of Chinese Medicine Wuhan Hubei Province China

**Keywords:** biomarker, diagnosis, FXYD6, glioma, prognosis

## Abstract

**Objective:**

As a single‐transmembrane protein of the FXYD family, FXYD6 plays different roles under physiological and pathological status, especially in the nervous system. This study aims to identify FXYD6 as a biomarker for glioma, by analyzing its expression and methylation patterns.

**Methods:**

Using TCGA and GTEx datasets, we analyzed FXYD6 expression in various tissues, confirming its levels in normal brain and different glioma grades via immunoblotting and immunostaining. FXYD6 biological functions were explored through enrichment analysis, and tumor immune infiltration was assessed using ESTIMATE and TIMER algorithms. Pearson correlation analysis probed FXYD6 associations with biological function‐related genes. A glioma detection model was developed using FXYD6 methylation data from TCGA and GEO. Consistently, a FXYD6 methylation‐based prognostic model was constructed for glioma via LASSO Cox regression.

**Results:**

FXYD6 was observed to be downregulated in GBM and implicated in a range of cellular functions, including synapse formation, cell junctions, immune checkpoint, ferroptosis, EMT, and pyroptosis. Hypermethylation of specific FXYD6 CpG sites in gliomas was identified, which could be used to build a diagnostic model. Additionally, FXYD6 methylation‐based prognostic model could serve as an independent factor as well.

**Conclusions:**

FXYD6 is a promising biomarker for the diagnosis and prognosis of glioma, with its methylation‐based prognostic model serving as an independent factor. This highlights its potential in clinical application for glioma management.

## 1 INTRODUCTION

FXYD domain‐containing ion transport regulator 6 (FXYD6) is a member of the FXYD family, which mainly contains homologous single transmembrane proteins FXYD1–FXYD7.^1^, ^2^, ^3^ The FXYD family members and the SPARC family members are known as the regulatory subunits of sodium‐potassium pump (Na,K‐ATPase, NKA).^3^, ^4^, ^5^, ^6^ FXYD6 is widely distributed in different tissues and organs, and related to different cancers, such as hepatocellular carcinoma, pancreatic cancer, and cholangiocarcinoma.^7^, ^8^, ^9^, ^10^ In nervous system, FXYD6 is found to be expressed in Type II taste bud cells,^11^, ^12^ Type II spiral ganglion neurons,^13^ two clusters of dorsal root ganglion (DRG) neurons,^14^ almost all subnuclei of parabrachial nucleus,^15^ as well as cerebellum and hippocampus.^16^, ^17^ According to single‐cell RNA‐sequencing, *Fxyd6* is mainly distributed in small‐diameter *Gal* cluster and *Th* cluster DRG neurons, the primary sensory neurons, suggesting the distinct functions of FXYD6 in somatosensation.^18^, ^19^ In fact, FXYD6 could bind to TRPV1 channel and increase capsaicin‐induced current in *Gal* cluster DRG neurons, thus promoting thermal nociception.^14^ Besides, some studies of FXYD6 have reported the specific candidate single nucleotide polymorphisms in schizophrenia,^20^, ^21^ the changed expression patterns in Alzheimer's disease (AD) Tg2576 mice,^22^ and the GWAS analysis in alcohol addiction.^23^ In a word, FXYD6 assumes significant functions in both physiological and pathological conditions, especially within the neurological system.

Nevertheless, there was limited knowledge regarding the role of FXYD6 in gliomas. Many studies have reported the role of different NKA alpha and beta subunits in gliomas, mainly involving the inhibition of NKA to trigger the cell death and inhibit glioma growth.^24^, ^25^, ^26^, ^27^ In addition, the potential roles of several FXYD family members in glioma have been reported, including FXYD2, FXYD3, and FXYD4.^28^, ^29^, ^30^ Futhermore, neural precursor cells could migrate to the glioma cells in a distance and further induce the cell death of glioma cells by stimulating the TRPV1 channel.^31^, ^32^, ^33^ Capsaicin and arvanil could activate TRPV1 to induce the glioma cell apoptosis via Ca^2+^‐entry and ER stress.^34^, ^35^ Considering that FXYD6 is a member of FXYD family and natural endogenous inhibitor of NKA, and could promote TRPV1 activity, therefore, we wondered about the possible role of FXYD6 in gliomas.

In this study, it was observed that FXYD6 had a widespread distribution in normal tissues, while experiencing a considerable downregulation in GBM samples. Specific FXYD6 CpG sites were screened by analyzing The Cancer Genome Atlas (TCGA) and Gene Expression Omnibus (GEO) data. Consistently, Kaplan–Meier (KM) curves showed that glioma patients with lower expression levels of FXYD6 or certain FXYD6 CpG sites with low methylation had bad prognosis. Hence, the present study suggests that FXYD6 holds potential as a valuable biomarker for the glioma diagnosis and prognosis.

## 2 METHODS

### 2.1 Data mining and databases

UCSC Xena was searched (http://xena.ucsc.edu/)^36^ to acquire RNA‐seq reads of tumor samples of the TCGA (https://www.cancer.gov/tcga.)^37^, ^38^ and normal tissues of GTEx database. Methylation data of FXYD6 CpG sites and glioma clinical data were obtained through TCGA database. And then through mRNAseq_693 dataset of Chinese Glioma Genome Atlas (CGGA) database,^39^ Rembrandt and Gravandeel (from Gliovis database, at http://gliovis.bioinfo.cnio.es/), expression and clinical data were downloaded.

According to 2021 WHO CNS tumor classification,^40^, ^41^ we collated the data in TCGA database, and no longer defined OA (Oligoastrocytoma) as a separate category, and it's also notable that WHO IV glioma consisted of Astrocytoma (IDH mutant, WHO Grade IV) and GBM (IDH wildtype).

Expression of FXYD6 in adult mouse brain was accessed from Allen Mouse Brain Atlas (https://mouse.brain‐map.org/static/atlas).^42^, ^43^ ARCHS4 database displayed the distribution of FXYD6 of mice (https://maayanlab.cloud/archs4/index.html). The methylation data of non‐glioma was obtained from the datasets of GEO database, at https://www.ncbi.nlm.nih.gov/geo/, and non‐glioma datasets for the diagnosis were composed of GSE168726, GSE147548, GSE108576, GSE40360. The glioma datasets to validate the effect of prognostic model in TCGA database consisted of GSE103659 and GSE48461 from GEO database. TIMER web server was applied to assess immune cell infiltration in glioma tissues (http://timer.cistrome.org/).^44^

### 2.2 Clinical samples

Patients with gliomas were recruited from the Department of Neurosurgery at Huashan Hospital affiliated to Shanghai Medical School, Fudan University from 2020 to 2022 for this study. The histological confirmation of glioma diagnosis was conducted in a blinded manner by a minimum of two experienced pathologists following surgical resection. Fresh biopsies of gliomas were collected at the time of surgical resection and were processed for immunoblotting. Cryostat sections of the tissues were processed for immunostaining.

### 2.3 Mice

C57BL/6J mice were purchased from Shanghai Laboratory Animal Center, Chinese Academy of Sciences (Shanghai, China). Mice were raised together with littermates in pathogen‐free environment and their health status was routinely checked.

### 2.4 Immunoblotting and immunostaining

The tissues processed for immunoblotting were lysed in ice‐cold tissue lysis buffer(150 mM NaCl, 30 mM HEPES, 10 mM NaF, 1% TritonX‐100, 0.01% SDS). The suspended lysate was incubated in sample buffer (50 mM Tris–HCl, pH 7.4, 2% SDS, 5% β‐mercaptoethanol, 10% glycerol, 0.01% bromophenol blue) for 5 min at 100°C. Then the immunoblotting was processed. The samples were loaded for SDS‐PAGE, transferred, probed with antibodies, and visualized with enhanced chemiluminescence (GE Amersham Imager600). The primary antibodies against FXYD6 (1:1000, Rb, Proteintech, SAB1101623, and 1:500, Rb, previous paper^14^), and Actin (1:100000, Mo, Chemicon, MAB1501), and corresponding horseradish peroxidase‐conjugated secondary antibody were used.

Glioma tissues were sectioned at five microns and fixed by 4% paraformaldehyde for 1 h. The sections were permeabilized and blocked by PBS with 0.2% Triton X‐100 and 5% bovine serum albumin. The frozen sections were incubated in primary antibodies against FXYD6 (1:2000, Rb, previous paper^14^) at 4°C overnight. After washing with PBS, the samples were stained with the secondary antibodies were Cy5‐conjugated donkey against rabbit (1:1000, Yeasen), and DAPI for 1 h at 37°C. The samples were visualized with a fluorescence microscope(TCS SP8 X, Leica).

### 2.5 Gene ontology (GO) analysis

Genes related to FXYD6 were identified by Pearson correlation analysis (*r* > 0.5, *p* < 0.05), and the average gene expression need be larger than 0.5. R package (enrichplot) analyzed FXYD6 related genes. The results were presented in the form of enrichment dot bubble.

### 2.6 Gene set variation analysis (GSVA)

Biological functions were considered to select genes through AmiGO2 portal (http://amigo.geneontology.org/amigo) to calculate the functional enrichment score of each glioma sample in the TCGA database by the given package (R environment).

### 2.7 Tumor microenvironment (TME) score evaluation and cell infiltration

We evaluated ImmuneScore, Stromalscore, and tumor purity computationally in RNA‐seq data of TCGA database using the ESTIMATE algorithm that applies gene expression signatures to deduce the fraction of stromal and immune cells in tumor samples. Then CIBERSORTx was used to estimate the proportions of the immune cell.^45^ By the deconvolution algorithm and a signature reference of immune cells (LM22), CIBERSORTx can accurately determine the immune infiltration of each sample. The expression data of gliomas in the TCGA database were put into the analysis.

### 2.8 The scRNA‐Seq data processing and analysis

We accessed the CGGA database to obtain scRNA‐Seq data,^46^ and processed the data in R language with R package “Seurat”. Through quality control, the function of FindVariableFeature could select the top 2000 highly variable genes. For these genes, principal component analysis (PCA) and Uniform Manifold Approximation and Projection (UMAP) were sequentially performed for dimensionality reduction and cluster identification. The function of FindAllMarkers was then applied when log2fc.threshold = 0.25 and min.pct = 0.25.

### 2.9 Statistical analysis

SPSS software (version 22·0 IBM), GraphPad Prism software (version 9·0), and R (version 4·2·2) were ustilized for statistical analyses and visualization. The difference between two groups was evaluated through unpaired *t* test. Correlation between two groups was assessed by Pearson correlation analysis. Multivariable logistic regression was used to construct the prediction model. Receiver operating characteristic (ROC) curves were plotted for the assessment of the diagnostic performance and KM was for the prognostic value. Univariate Cox regression analysis was performed for FXYD6 CpG sites in TCGA database. CpG sites with *p* < 0.05 was included in least absolute shrinkage and selection operator (LASSO) Cox regression. LASSO regression modeling was conducted using the R package, glmnet.^47^ Univariate Cox regression assessed the significance of the parameters of prognostic value, and variables would be included in the multivariate Cox regression, provided *p* < 0.2. The results were considered relevant and statistically significant when *p* < 0.05.

## 3 RESULTS

### 3.1 FXYD6 is widely distributed in normal tissues and downregulated in GBM

**FIGURE 1:**
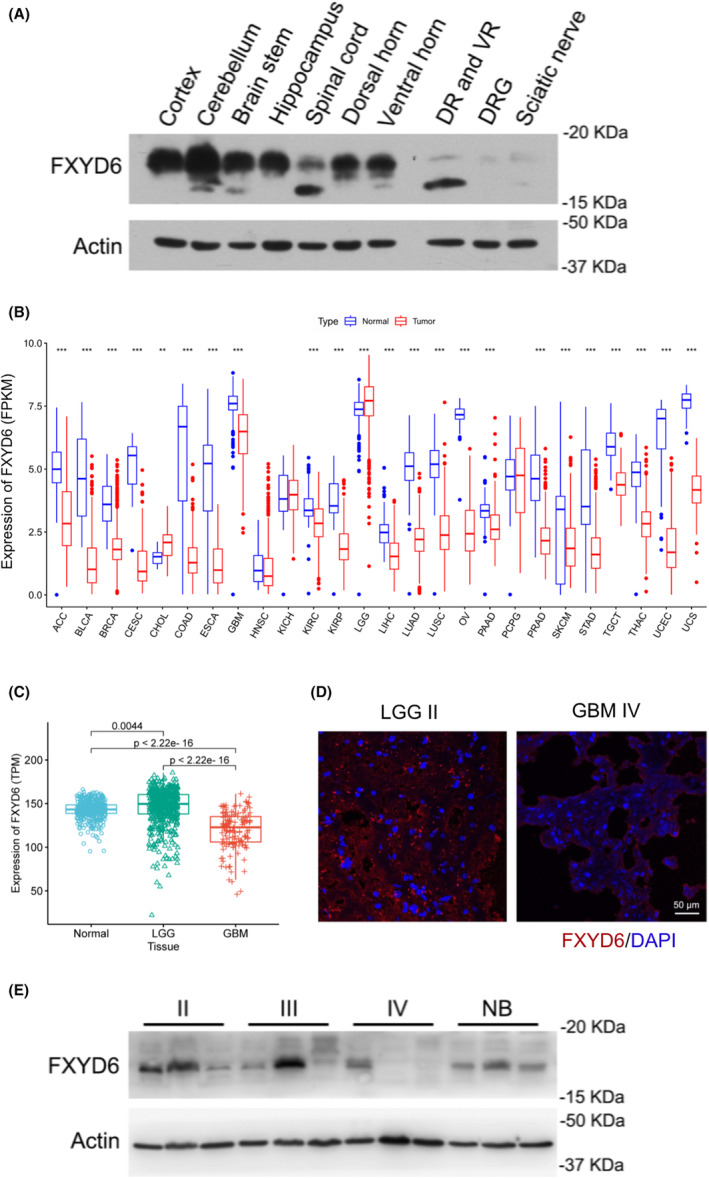
FXYD6 expressed highly in brain tissues and down‐regulated in GBM. (A) Various parts of CNS tissues and PNS tissues from mice were collected for western blot. (B) FXYD6 expression profiling showed extensive expression and special enrichment in brain tissues. (C) FXYD6 expression in normal brain tissues (*n* = 423), LGG (*n* = 529), and GBM (*n* = 139) was compared via the integrated dataset from the TCGA database and GTEx. FXYD6 was slightly increased in LGG, but significantly down‐regulated in GBM. (D) Immunofluorescence data showed that FXYD6 was lower in GBM than LGG samples. (E) Western blot showed FXYD6 expression of grade II and III gliomas reached similar expression level of normal brain tissues, all of which were far more than those in GBM.

Firstly, FXYD6 was widely distributed but varied greatly in diverse normal tissues, through the online database (Figure S1A). Obviously, it was mainly enriched in central nervous system (CNS) and peripheral nervous system (PNS) compared with other tissues. Additionally, western blot revealed the expression of FXYD6 in mice, which was more expressed in CNS, such as cortex, cerebellum, than PNS (DR and VR, DRG, sciatic nerve) (Figure 1A). In situ hybridization visually demonstrated the high expression of FXYD6 in the hippocampus, cerebellum, and cerebral cortex, according to Allen Brain Atlas database (Figure S1B).

Similarly, through the combined analyses of TCGA and the Genotype‐Tissue Expression (GTEx), expression profilings of FXYD family members showed the distribution of FXYD1‐FXYD6 in diverse cancers and normal tissues in human. On the one hand, FXYD1 was downregulated in almost all cancers indicating a promising pan‐cancer biomarker in the future, while FXYD2‐FXYD5 failed to exhibit the specific tendency in gliomas compared with other cancers (Figure S2). On the other hand, distinctively, FXYD6 was extensively expressed and especially enriched in brain tissues no matter in gliomas or normal tissues (Figure 1B). Additionally, FXYD6 was increased slightly in lower‐grade glioma (LGG), but significantly downregulated in glioblastoma (GBM) (Figure 1C). As expected, immunofluorescence data demonstrated that FXYD6 was significantly decreased in GBM, compared to LGG (Grade II) (Figure 1D). Identically, western blot also showed FXYD6 protein levels both in LGG (Grades II and III) and normal brain tissues were higher than those in Grade IV gliomas (Figure 1E).

### 3.2 FXYD6 expression level is correlated with molecular and pathological features of glioma

**FIGURE 2:**
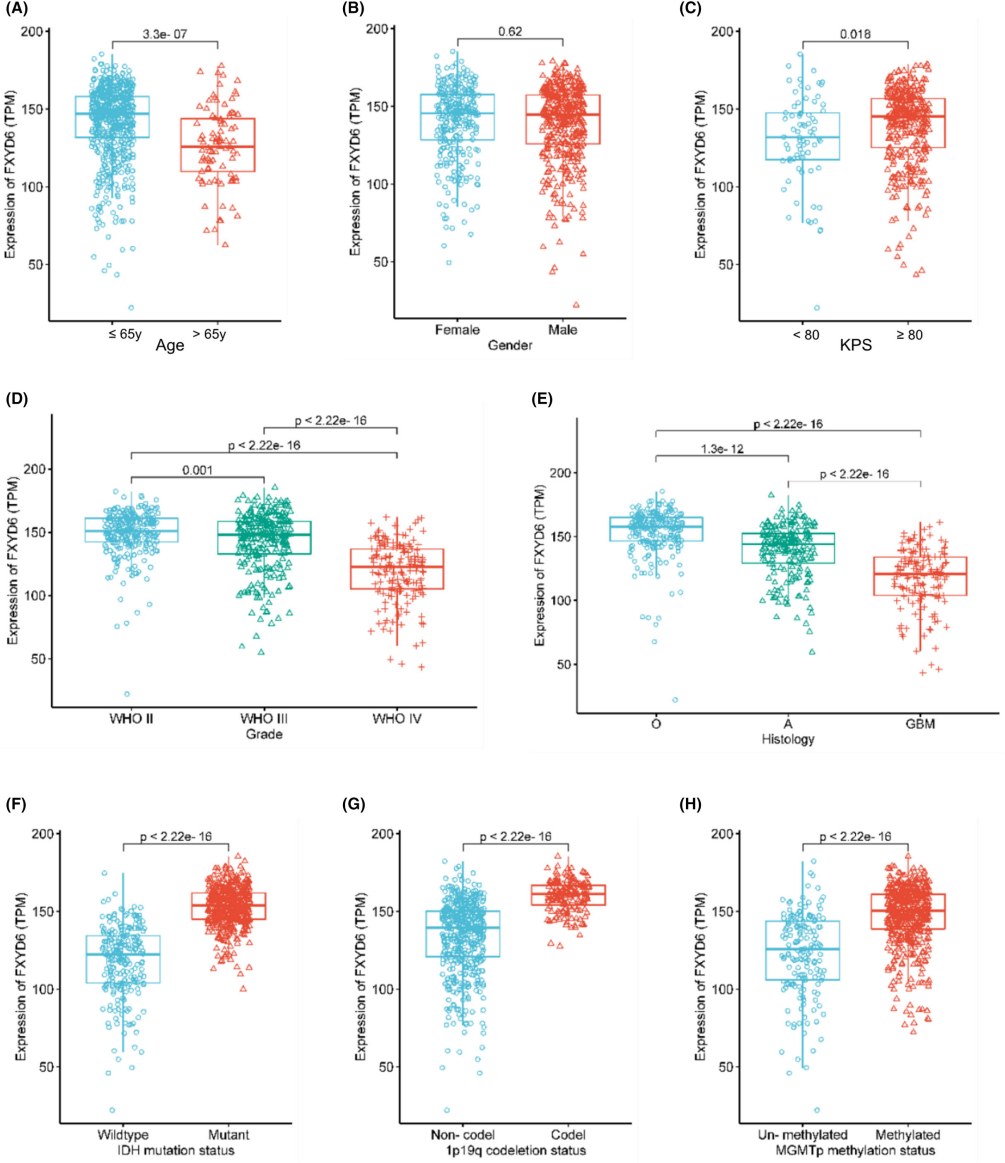
FXYD6 expression in gliomas was generally correlated with clinical features according to the TCGA database. (A) Elderly patients (age > 65 years) (*n* = 89) showed a lower FXYD6 expression than younger patients (age ≤ 65 years) (*n* = 601). (B) FXYD6 expression was impertinent to the gender, female (*n* = 295), male (*n* = 395). (C) Patients with KPS ≥80 had higher FXYD6 expression, KPS < 80 (*n* = 75), KPS ≥80 (*n* = 352). (D) Expression of FXYD6 decreased with the elevated grade, Grade II (*n* = 257), Grade III (*n* = 266), Grade IV (*n* = 167). (E) FXYD6 expression was highest in oligodendroglioma (O) and the lowest in GBM, O (*n* = 288), A (*n* = 203), GBM (*n* = 139). (F–H) FXYD6 was significantly less expressed in IDH wildtype (*n* = 233) than IDH mutant (*n* = 423) gliomas, in without 1p/19q codeletion (*n* = 490) than 1p/19q codeletion (*n* = 168), in un‐methylated MGMTp (*n* = 166) than methylated MGMTp.

The expression of FXYD6 in gliomas demonstrated a general association with the cliniopathological characteristics of gliomas, as observed in TCGA database. First, elderly patients showed lower FXYD6 expression (Figure 2A), but it was impertinent to the gender. Meanwhile, when Karnofsky Performance Status (KPS) ≥ 80, patients showed higher FXYD6 expression (Figure 2C). Additionally, it decreased with the elevated grades (Figure 2D). Meanwhile, histology seemed related to FXYD6 expression, which was highest in Oligodendroglioma (O) and lowest in GBM (Figure 2E). The distribution of FXYD6 in gliomas futher displayed the down‐regulation in IDH wildtype (Figure 2F), 1p/19q non‐codeletion (Figure 2G), and un‐methylated MGMTp (Figure 2H).

### 3.3 FXYD6 is associated with various immune functions, EMT, ferroptosis and pyrocytosis

**FIGURE 3:**
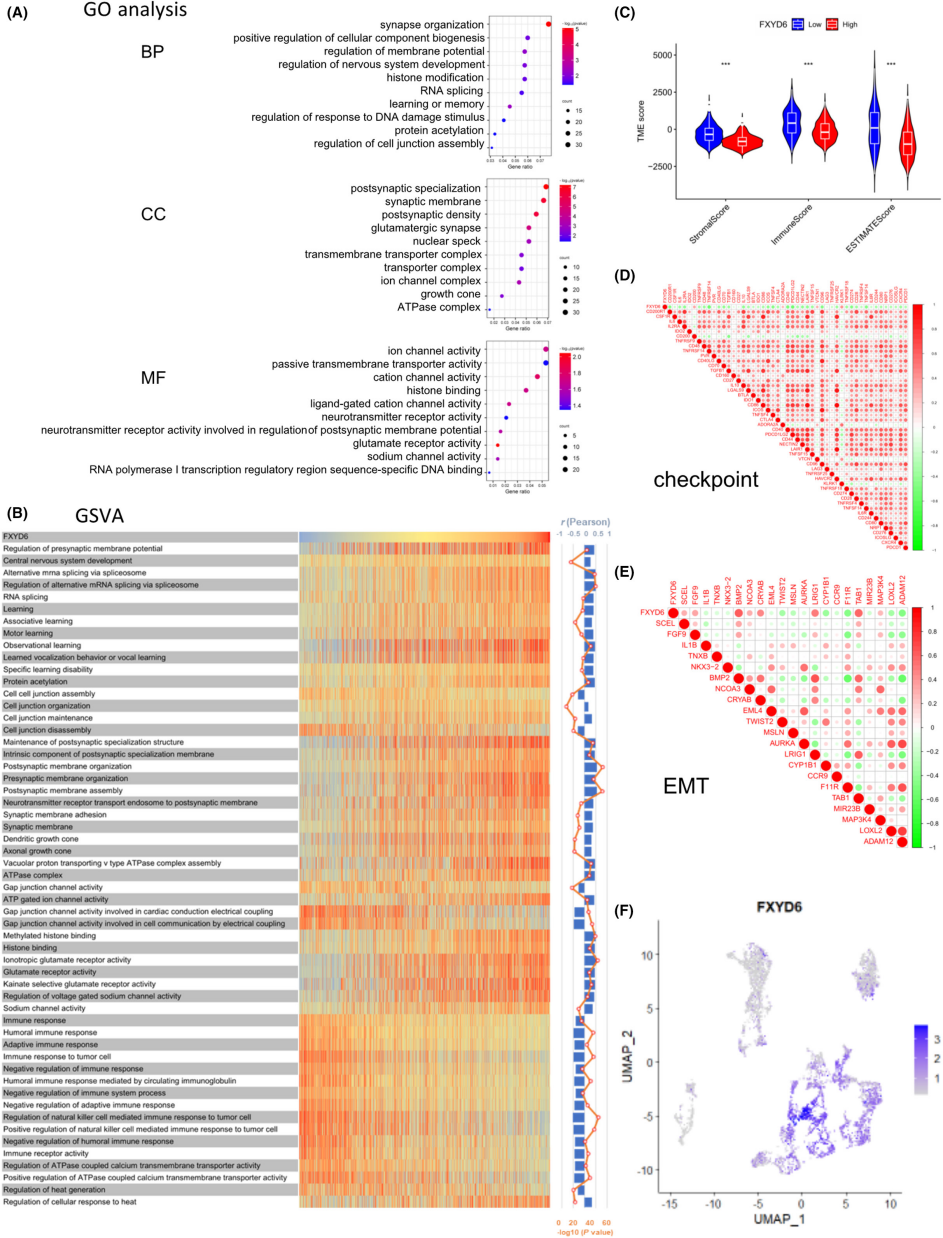
Biological functions of FXYD6 were analyzed comprehensively. (A) In TCGA database, FXYD6 related genes were analyzed through GO enrichment analysis. (B) The relationship between FXYD6 expression and various biological functions was analyzed by GSVA. (C) TME violin plots showed glioma tissues relationship of FXYD6 with TMEscore. (D, E) Correlation matrix showed the relationship of FXYD6 with immune checkpoints and EMT in gliomas. (F) In the analysis of scRNAseq, it showed the clusters of FXYD6 in glioma tissue cells.

FXYD6 related genes in TCGA database was then utilized to perform GO analysis (Figure 3A). The biological process of FXYD6 has been implicated in the organization of synapses, the positive regulation of cellular component biogenesis, and the regulation of membrane potential. FXYD6 related cell component was chiefly in synaptic‐related components. Molecular functions were ion channel activity, passive transmembrane transporter activity, cation channel activity, and so forth. Next, the above biological and immunological functions were then included in GSVA with TCGA data (Figure 3B). The findings indicated FXYD6 was connected with the RNA splicing, cell junction organization and maintenance, synaptic membrane organization, histone binding, and various immunological functions in gliomas.

The estimation of the proportion of immunity and stroma was conducted using the expression of FXYD6 in TCGA database. Clearly, glioma samples with high expression of FXYD6 had lower stromascore, immunescore, and estimatescore (Figure 3C), which indicated FXYD6 expression was negatively correlated with the amout of immune and stromal proportion. Meanwhile, for low expression of FXYD6 there was higher fraction of B cells naïve, CD8 T cells, and macrophages M1, yet less NK cells resting, and monocytes in gliomas (*p* < 0.05) (Figure S3A).

Then with glioma samples in TCGA database, FXYD6 expression was negatively correlated with the infiltration of macrophages M1, CD8 T cells and positively with monocytes (Figure S3B). By validation of TIMER2.0 database, the expression of FXYD6 was mainly negatively correlated with the infiltration of multiple immune cells in both GBM and LGG samples (Figure S3C–D).

Next, the relationship of FXYD6 expression with the functional states in gliomas was explored, for instance, immune checkpoints, ferroptosis, EMT and pyroptosis. First, the expression of FXYD6 was negatively correlated with most of immune checkpoints, especially the immune checkpoint inhibitors, such as PDCD1LG2, PDCD1, IDO1, and so on. (Figure 3D). Undountedly, these results revealed that FXYD6 expression had a strong association with the activation of immunological functions to gliomas. Second, FXYD6 expression was also closely relevant to ferroptosis genes. Remarkably, it was positively correlated with drivers of ferroptosis and negatively with suppressors (Figure S3E), implying FXYD6 might promote ferroptosis in gliomas which supported that the decreased immunological infiltration with the increase of FXYD6. Moreover, FXYD6 was pertinent to the several classic EMT genes in gliomas (BMP2, TAB1, and so on) (|*r*| > 0.35, *p* < 0.05) (Figure 3E). It also revealed that FXYD6 expression was negatively correlated with the majority of pyroptosis genes (Figure S3F).

The scRNAseq dataset of CGGA database was then analyzed and the cells were divided into 18 clusters (Figure S3G). Obviously, FXYD6 was mainly enriched in the clusters of 2, 4–8, 10–12, and 16, which represented the distribution of stem cells of gliomas^48^ and neuron,^49^ showing FXYD6 was generally related to these cells in gliomas.

Overall, FXYD6 was enriched in glioma stem cells and neuron, highly relevant to RNA splicing, cell junction, and various immunological functions in gliomas. Additionally, it might be implicated in the suppression of inhibitory immune checkpoints, and pertinent to the ferroptosis, EMT and pyroptosis in gliomas.

### 3.4 Glioma patients with low expression levels of FXYD6 have a bad prognosis

**FIGURE 4:**
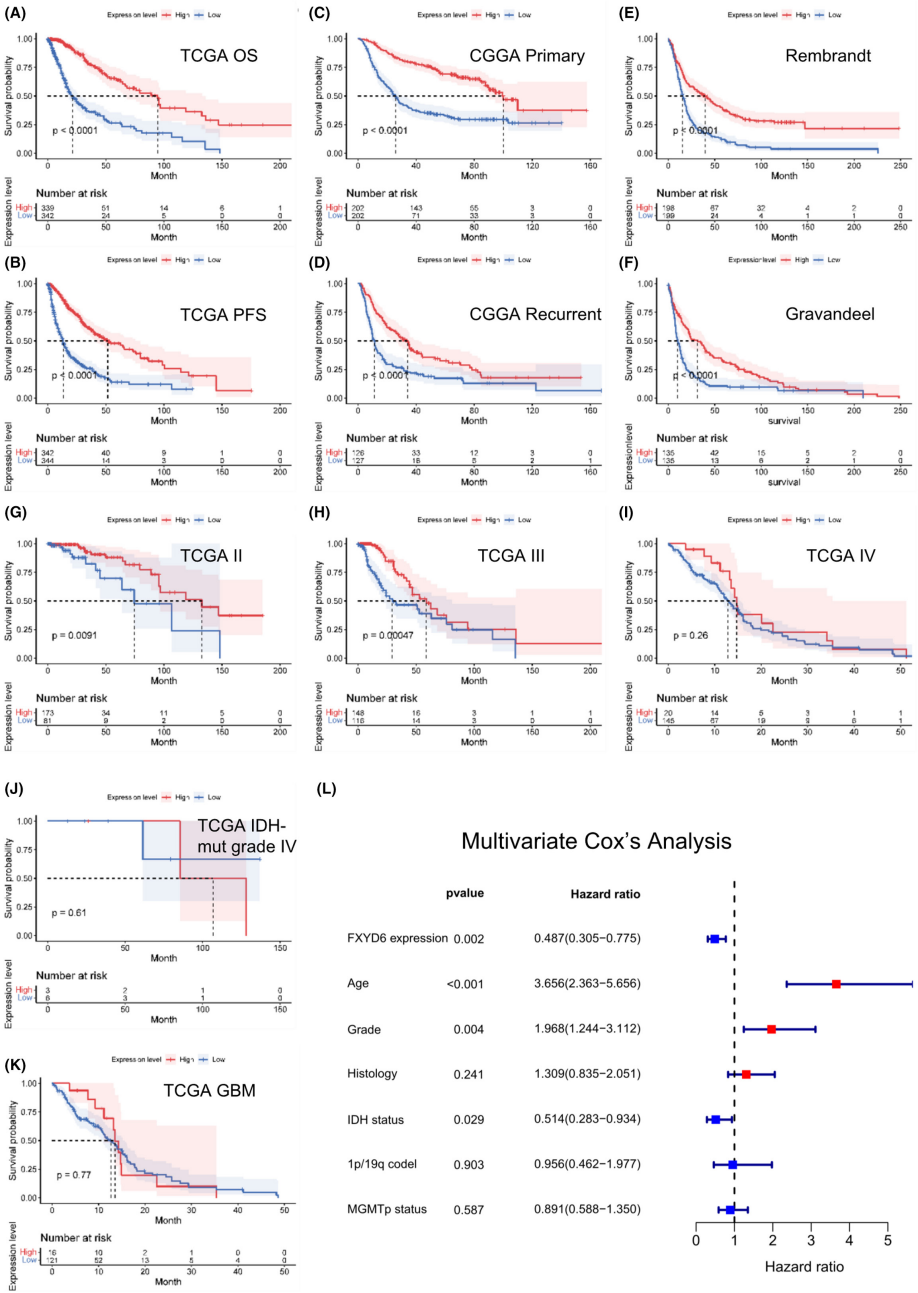
FXYD6 expression was correlated with prognosis of glioma patients through multiple databases. (A, B) KM analysis of OS and PFS in gliomas in TCGA database. (C, D) KM curve of FXYD6 expression was plotted in primary and recurrent glioma in CGGA database. (E, F) KM analysis of OS in gliomas in Rembrandt and Gravandeel database (G–I) In WHO II‐IV gliomas, KM curves showed OS in high and low FXYD6 expression. (J, K) In TCGA database, KM curves of OS in IDH‐mutant Grade IV and GBM (IDH wildtype). (L) Multivariate Cox's analysis of common prognostic parameters in TCGA database was performed.

KM analysis was then conducted by public databases to explore the prognostic value of FXYD6. Glioma patients with higher FXYD6 expression showed significantly longer overall survival (OS) and progression‐free survival (PFS) than those with lower FXYD6 expression in TCGA database (Figure 4A,B). Similaly, patients with high expression of FXYD6 had longer OS in primary and recurrent glioma patients of CGGA database (Figure 4C,D). Rembrandt and Gravandeel databases also confirmed the same results (Figure 4E,F). In TCGA database, KM curves showed statistically significant survival differences in WHO II and III gliomas but not Grade IV (Figure 4G‐I). According to 2021 WHO CNS tumor classification,^40^ similarly, we still did not find statistical significance of OS about different FXYD6 expression in Astrocytoma (IDH mutant, WHO Grade IV) and GBM (IDH wildtype).

Furthermore, Cox regression analysis showed FXYD6 expression was an independent prognostic factor for the glioma patients, including various common prognostic items.

### 3.5 FXYD6 CpG site candidates could construct the diagnostic model

**FIGURE 5:**
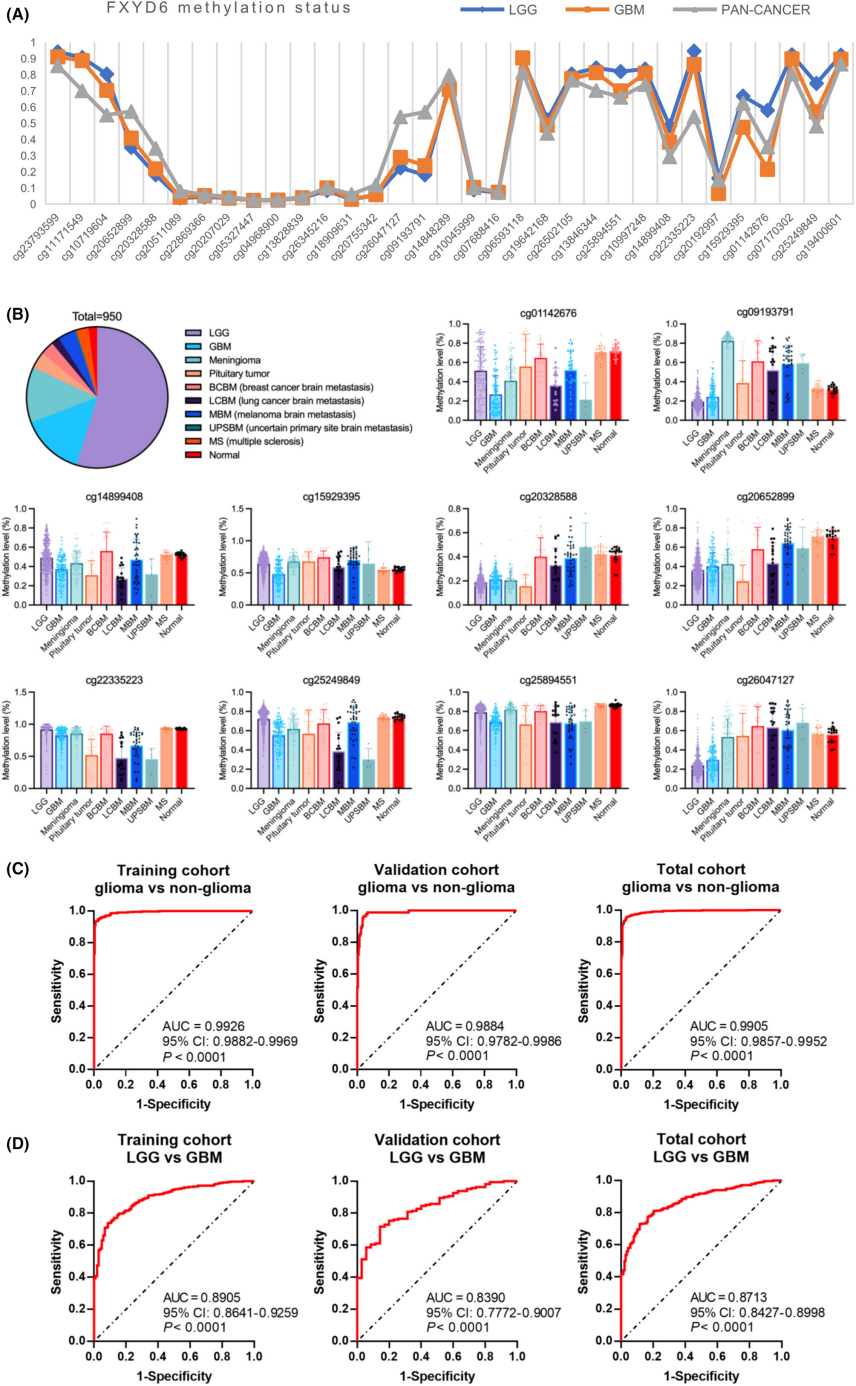
Diagnostic models were established based on methylation levels of selected FXYD6 CpG sites. (A) According to TCGA database, methylation levels of FXYD6 in LGG, GBM and pan‐cancers were plotted. (B) The proportion of various brain tissues was shown. And the methylation level of various tissues at the 10 selected FXYD6 DNA CpG sites. (C, D) ROC curves constructed by selected FXYD6 CpG sites to differentiate gliomas from non‐gliomas and LGG from GBM.

Specific CpG sites of some genes were screened in glioma, and further applied into the diagnosis and prognosis, like SHOX2 and MAL2.^50^, ^51^ To construct a detection model for gliomas, the difference of methylation at FXYD6 CpG sites between pan‐cancers and LGG/GBM need be larger than 0.2. Finally, 10 FXYD6 CpG sites were selected to distinguish gliomas (Figure 5A). Methylation difference of FXYD6 at selected CpG sites between different brain tissues were presented (Figure 5B). Obviously, cg09193791, cg22335223 and cg26047127 performed well in the detection of gliomas (Figure S4A). And cg01142676, cg15929395, cg22335223, cg25249849, and cg25894551 did effectively to distinguish LGG from GBM (Figure S4B).

To obtain a more effective detection model to distinguish gliomas, multivariable logistic regression was used with the selected CpG sites (Figure 5C). In the training cohort (*n* = 665), ROC curve showed at the optimal cut‐off value of 0.8056, the sensitivity reached 93.6% with the specificity of 99.0%. In the validation cohort (*n* = 285), at the same cut‐off value, the diagnostic model showed a sensitivity of 77.1% and a specificity of 99.0%. Congruously, the total cohort, at the best cut‐off value of 0.6488, presented a sensitivity of 95.7% and a specificity of 96.6%.

The diagnostic model for distinguishing LGG (*n* = 520) from GBM (*n* = 138) also exhibited relatively excellent performance (Figure 5D). Training cohort achieved the sensitivity of 73.7% and specificity of 91.3% at the optimal cutoff value of 0.8744, at which the validation cohort acquired the sensitivity of 69.0% and specificity of 85.7%. Specially, in the total cohort, ROC curve showed a sensitivity of 72.6% with the specificity of 88.4% at the best cut‐off value of 0.8595.

Overall, the diagnostic model based on the methylation of FXYD6 CpG candidates could distinguish gliomas from non‐gliomas and LGG from GBM relatively accurately and stably.

### 3.6 The FXYD6 methylation‐based risk score acts as an prognostic adverse factor to guide the therapy of gliomas

**FIGURE 6:**
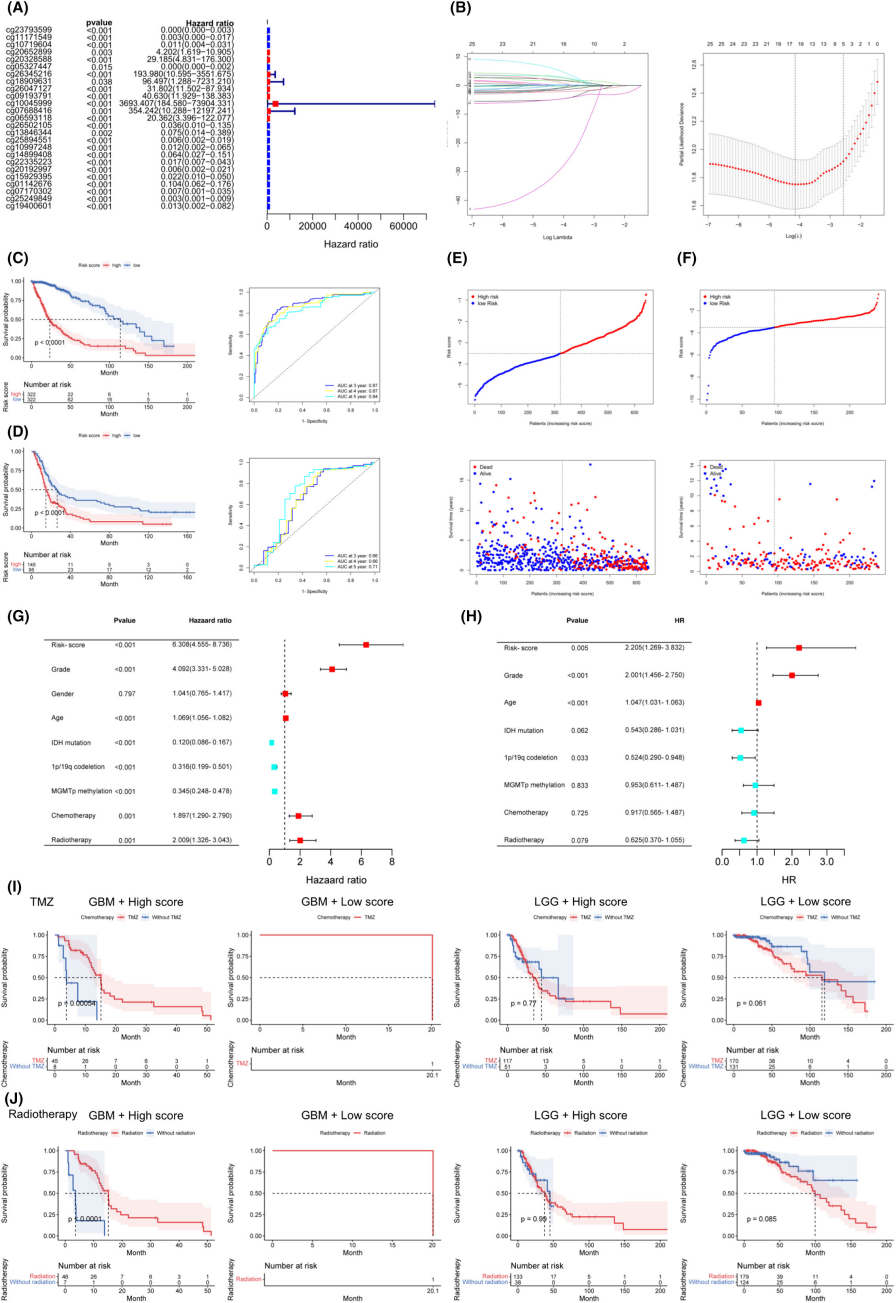
Risk‐score models constructed by methylation of FXYD6 have critical prognostic values. (A) Univariate Cox regression for all the FXYD6 CpG sites recorded in TCGA database. (B) The selected CpG sites were included into LASSO Cox reguression to construct the risk‐score model. (C, D) KM curve and ROC time‐dependent curve of risk‐score model for prognosis of glioma patients in the training cohort and the validation cohort. (E, F) Risk plot showed prognostic tendency in the training cohort and the validation cohort. (G, H) Univariate and multivariate Cox regression for the risk‐score model and common clinicopathological characteristics. (I, J) The risk‐score model could guide the chemotherapy and radiotherapy for LGG/GBM with high and low risk.

In total, 25 CpG sites of FXYD6 recorded in TCGA database were displayed, whose *p* value was below 0.05 (Figure 6A). Subsequently, these CpG sites constructed a risk‐score model (Figure 6B). TCGA database acted as the training cohort (*n* = 644), and GEO database as the validation cohort (*n* = 240). In the training cohort, low‐risk glioma patients presented longer median survival than high‐risk glioma individuals, and the risk‐score model could predict the prognosis accurately (Figure 6C). The excellent performance was confirmed in the validation cohort (Figure 6D). Additionally, with the increase of risk‐score, there was an increase in the number of deaths and decrease in OS in both the training and validation cohort (Figure 6E,F).

Next, univariate Cox regression was carried out for clinical parameters (Figure 6G). The items with *p* < 0.05 were then included into multivariate Cox regression, and risk‐score model, *p* = 0.005, the hazard risk: 2.205 (Figure 6H). Additionally, the risk‐score model could also guide the chemotherapy (Figure 6I) and radiotherapy (Figure 6J) for glioma patients. High‐risk GBM patients with temozolomide (TMZ) had longer OS than those without TMZ. There was only one low‐risk GBM patient, who took TMZ. High‐risk and low‐risk LGG patients with TMZ failed to show different OS compared with those without TMZ. Identically, high‐risk GBM patients with radiotherapy had longer OS. The number of GBM with low‐risk score was merely one. Besides, High‐risk and low‐risk LGG patients accepting radiotherapy did not reaveal significant difference with not accepting radiotherapy.

## 4 DISCUSSION

The present study showed that FXYD6 was a novel biomarker for gliomas. For years, FXYD6 had already been discovered to be associated with NKA^3^ and extensively expressed in most of tissues in human which was consistent with the results of expression profiling. As a member of classical and critical NKA regulator FXYD family, changes of FXYD6 expression were also found to be related to the progression of various cancers.^8^, ^9^ Recently, one study applied the scRNA‐seq to characterize the heterogeneity response to inflammation of different astrocyte subpopulations [15], demonstrating that Fxyd6, as well as Thbs4, Igfbp5, are the synaptogenic markers, which are highly expressed in Cluster 6.^52^, ^53^ In this study, the enrichment of FXYD6 in CNS reminded us the pivotal role of it in the functions of nervous system, and we discovered down‐regulation of FXYD6 was pertinent to the growth and tumor microenvironment of glioma, especially GBM.

Generally, downregulation of FXYD6 could act as an unfavorable biomarker for prognosis in gliomas. Meanwhile, FXYD6 expression was germane to its methylation, and thus we looked forward to constructing the diagnostic model to distinguish gliomas. First, there was indeed substantial difference of methylation level between pan‐cancers and LGG/GBM at certain CpG sites. These FXYD6 CpG candidates were expected to detect gliomas. Additionally, some CpG sites whose *p* value were below 0.05 according to univariate Cox regression, managed to build a risk‐score model to serve as an independent adverse factors and guide the treatment of gliomas.

In order to fathom the reasons for the bad prognosis of glioma patients with down‐regulation of FXYD6, we further explored the biological functions of FXYD6 in gliomas. Through GO analysis and GSVA, FXYD6 was not only correlated with RNA splicing, cell junction organization and maintenance, synaptic membrane organization, histone binding, but multiple immunological functions in gliomas. Additionally, FXYD6 expression was generally negatively correlated with many classic inhibitory immune checkpoints, which suggested down‐regulation of FXYD6 might be involved in the inhibition of immune response to glioma cells. Meanwhile, the expression of FXYD6 was also relevant to ferroptosis, EMT and pyroptosis genes, all of which were possible to exert an effect on the progression of gliomas.

Interestingly, low expression of FXYD6 in LGG tend to have a longer OS, but it failed to show statistical significance in GBM. This phenomenon may be related to the following reasons. First, Grade IV gliomas tend to indicate more complications and shorter survival time. As a result, although we have observed a slight trend of difference of OS in Grade IV gliomas, there is no statistical significance. But on the other hand, LGG patients generally have a longer survival, making it possible that FXYD6 affects immune‐related functions to improve patient outcomes. Furthermore, according to our previous studies, FXYD6 co‐expresses with TRPV1 in Gal^+^ type small‐diameter DRG neurons, and could interact with TRPV1 channel by the highly negatively charged C‐terminal PGDEE motif, and thus to increase its capsaicin‐sensitive currents in mice.^14^, ^18^ As to TRPV1, the one 2021 Nobel molecule, it was studied well and most known for its role in somatosensation, especially in noxious heat.^54^ In addition, stimulation of TRPV1 could activate ATF3‐ER stress pathway, and help neural precursor cells to induce the cell death of high‐grade astrocytomas.^32^ Taken together, there is a possbile role of FXYD6 in gliomas by interacting with and regulating TRPV1 channel, which needs to be further studied comprehensively, via overexpressing the FXYD6 and/or TRPV1 in GBM cell lines to confirm that whether FXYD6 could affect the cell status, including cell death, apoptosis, and so on.

NKA consists of different catalytic alpha subunits, auxiliary beta subunits, and regulatory FXYD subunits, and is widely distributed in a subunit‐specific manner. Meanwhile, NKA was related in cancers and gliomas, by altering the cell status, including cell death and growth.^24^, ^25^, ^27^ Its subunits were also found to be crucial for cancers, including FXYD3, FXYD4, FXYD5 etc.^28^, ^29^ Due to FXYD6 is an unnecessary subunit of NKA, and could regulate its activity, therefore, we speculated that FXYD6 could change the cell status of GBM cell by regulating NKA.

In summary, FXYD6 is downregulated in GBM, and could act as a biomarker of prognosis of glioma patients. Methylation risk‐based model of FXYD6 could guide the treatment of glioma patients with TMZ and radiotherapy, thus making a contribution to health of human.

## AUTHOR CONTRIBUTIONS

**Weiliang Hou:** Methodology (equal); writing – original draft (equal); writing – review and editing (equal). **Jing Cai:** Methodology (equal). **Pei Shen:** Methodology (equal). **Shuo Zhang:** Methodology (equal). **Siyu Xiao:** Methodology (supporting). **Pu You:** Methodology (supporting). **Yusheng Tong:** Methodology (supporting). **Kaicheng Li:** Conceptualization (equal); writing – review and editing (equal). **Zengxin Qi:** Conceptualization (equal); funding acquisition (equal); writing – review and editing (equal). **Hao Luo:** Conceptualization (equal); writing – original draft (equal); writing – review and editing (equal).

## FUNDING INFORMATION

This study was supported by grants from the National Natural Science Foundation of China (81572483, 82072785, and 82272116), Shanghai Municipal Science and Technology Major Project (2018SHZDZX01) and ZJLab; Science and Technology Commission of Shanghai Municipality (20Z11900100, and 20S11905600); MOE Frontiers Center for Brain Science; Shanghai Shenkang (SHDC2020CR3073B); SHANGHAI ZHOU LIANGFU MEDICAL DEVELOPMENT FOUNDATION “Brain Science and Brain Diseases Youth Innovation Program”.

## CONFLICT OF INTEREST STATEMENT

All authors declare that we have no conflict of interest.

## ETHICS STATEMENT

Approval of the research protocol by an institutional review board: The study protocol was approved by The Institutional Review Board Huashan Hospital, Fudan University, and the whole research conforms to the provisions of the Declaration of Helsinki.

## INFORMED CONSENT

Written informed consent was achieved from each patient.

## ANIMAL STUDIES

All animal experiments were approved by the Institutional Animal Care and Use Committee.

## Supporting information


Figure S1.

## Data Availability

The data supporting this study are available within the article, supplementary information, or are publicly available.
