# Supplementary material for: Identification of FXYD6 as the novel biomarker for glioma based on differential expression and DNA methylation
Source: Cancer Med. 2023 Dec 13;12(24):22170–84. doi: 10.1002/cam4.6752 (PMC10757084; doi:10.1002/cam4.6752)

**Identification of FXYD6 as the novel biomarker for glioma based on differential expression and DNA methylation**

Weiliang Hou^1,#^, Jing Cai^1,#^, Pei Shen^3,#^, Shuo Zhang^2,#^, Siyu Xiao^4^, Pu You^2^, Yusheng Tong^1^, Kaicheng Li^2,^*, Zengxin Qi^1,^*, Hao Luo^2,^*

**Supplementary figure legends 1-4**

**Supplementary figures 1-4**

**Figure S1.** FXYD6 was widely expressed and enriched in CNS specially. (a) ARCHS4 database showed FXYD6 in human was extensively distributed in diverse normal tissues and organs of the body, but varied greatly. It was highly expressed in CNS and PNS compared with other parts of the body. (b-c) Expression data from Allen Mouse Brain Atlas visually demonstrated the distribution of FXYD6 in adult mouse brain tissues. Mouse.brain-map.org/experiment/show/73592534 (coronal), and mouse.brain-map.org/experiment/show/69113656 (sagittal).

**Figure S2.** Expression profiling showed the expression of FXYD1-5 in common cancers and corresponding normal tissues.

**Figure S3.** FXYD6 was correlated with immune functions, ferroptosis and pyroptosis. (a) There was higher fraction of B cells naïve, CD8 T cells, and macrophages M1, yet less NK cells resting, and monocytes in gliomas with low expression of FXYD6 (*P* < 0.05) (b) FXYD6 expression was negatively correlated with the infiltration of macrophages M1, CD8 T cells, T cells gamma delta, B cells naïve and positively with monocytes. (c) In the TIMER 2.0 database, the relationship of FXYD6 expression with the infiltration of immune cells in GBM samples was plotted. (d) In the TIMER 2.0 database, the relationship of FXYD6 expression with the infiltration of immune cells in LGG samples was plotted. (e) Correlation matrix showed the relationship of FXYD6 expression with ferroptosis genes. (f) Correlation matrix showed the relationship of FXYD6 expression with pyroptosis. (g) 6,148 cells in 73 regions from 14 patients of ScRNAseq dataset in CGGA database contained were then divided into 18 clusters.

**Figure S4.** ROC curves to distinguish gliomas from non-gliomas and LGG from GBM at different FXYD6 DNA CpG sites. (a) ROC curves to separate gliomas from non-gliomas at the selected FXYD6 CpG sites. (b) ROC curves to separate LGG from GBM at the selected FXYD6 CpG sites.


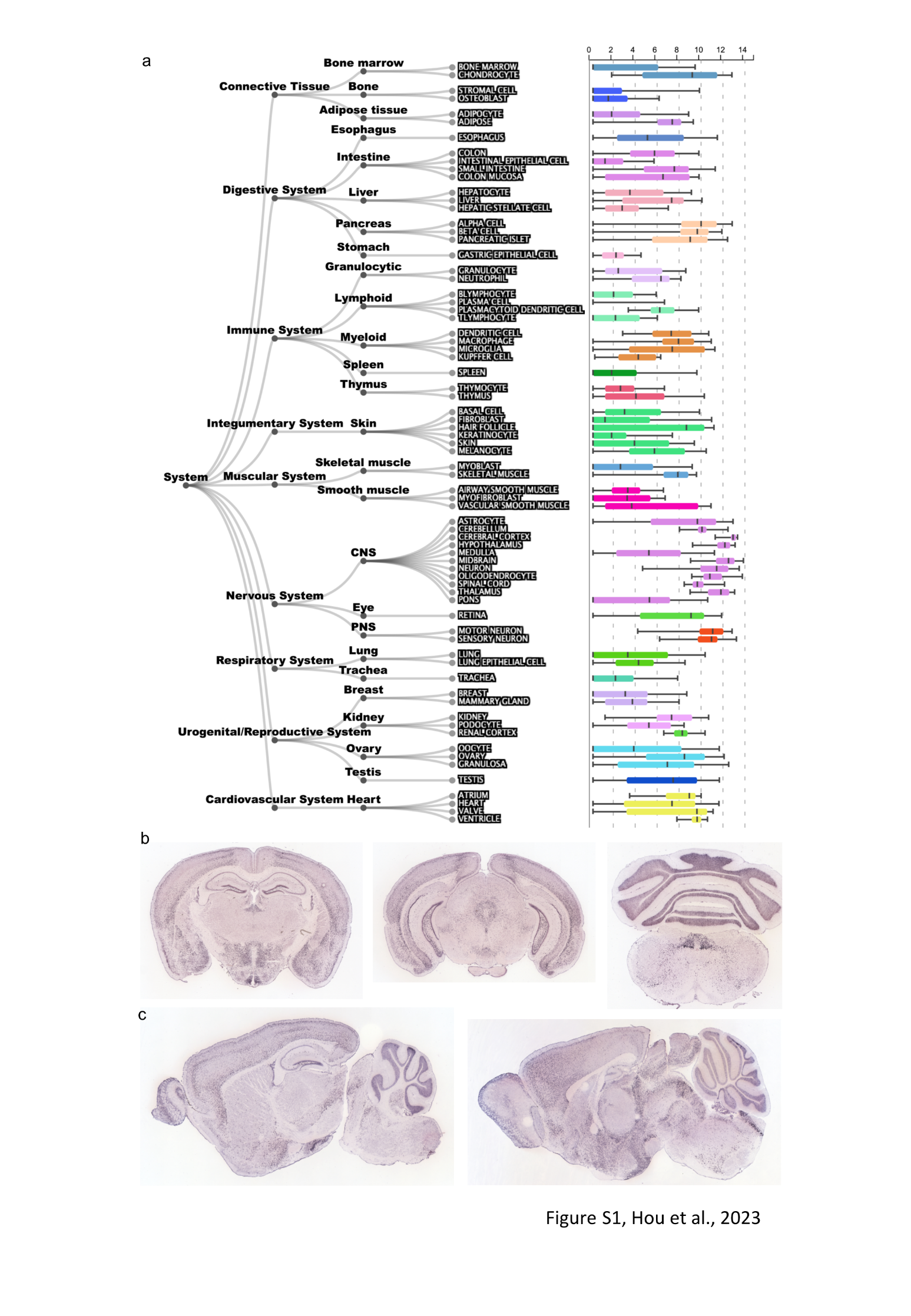

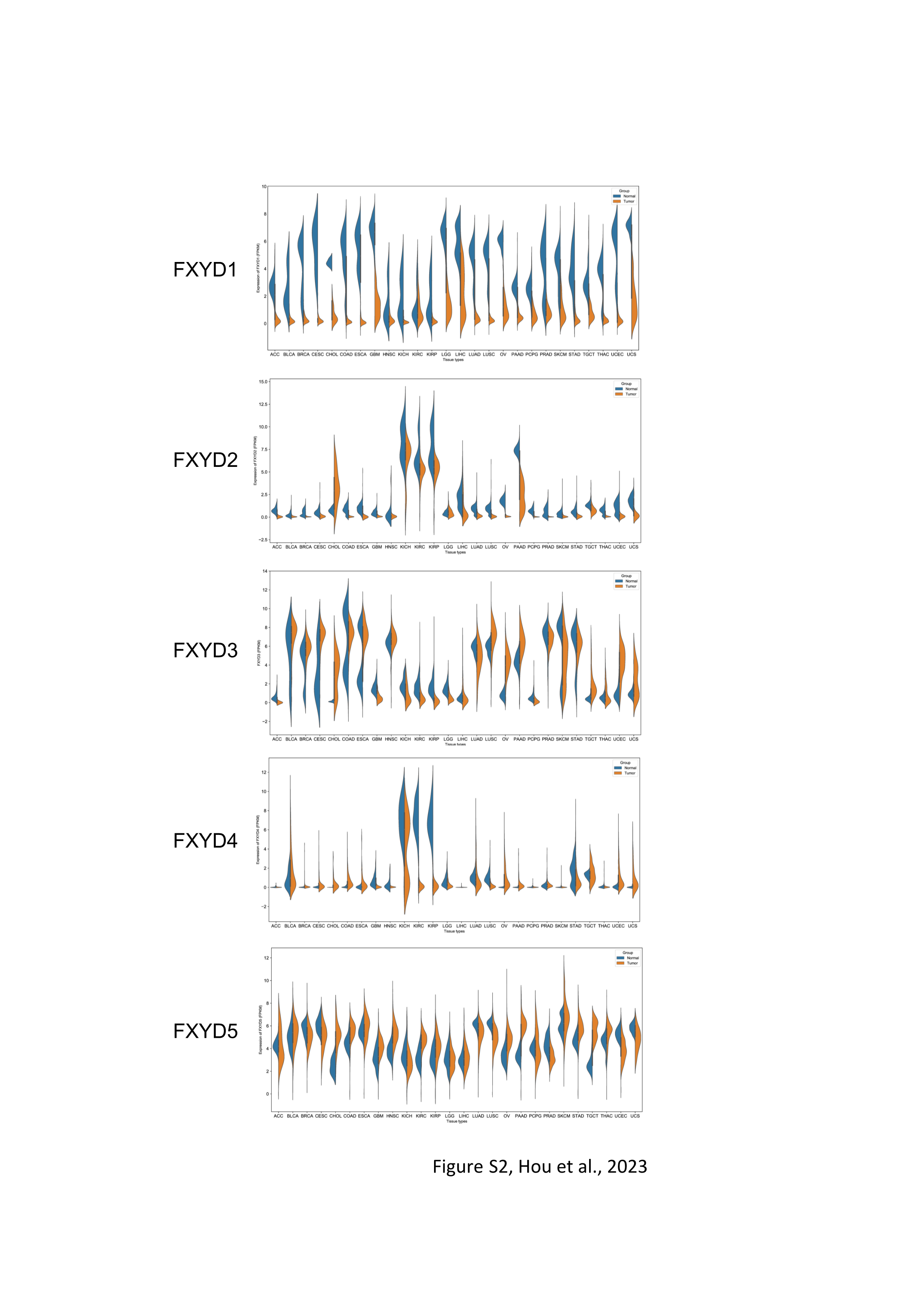

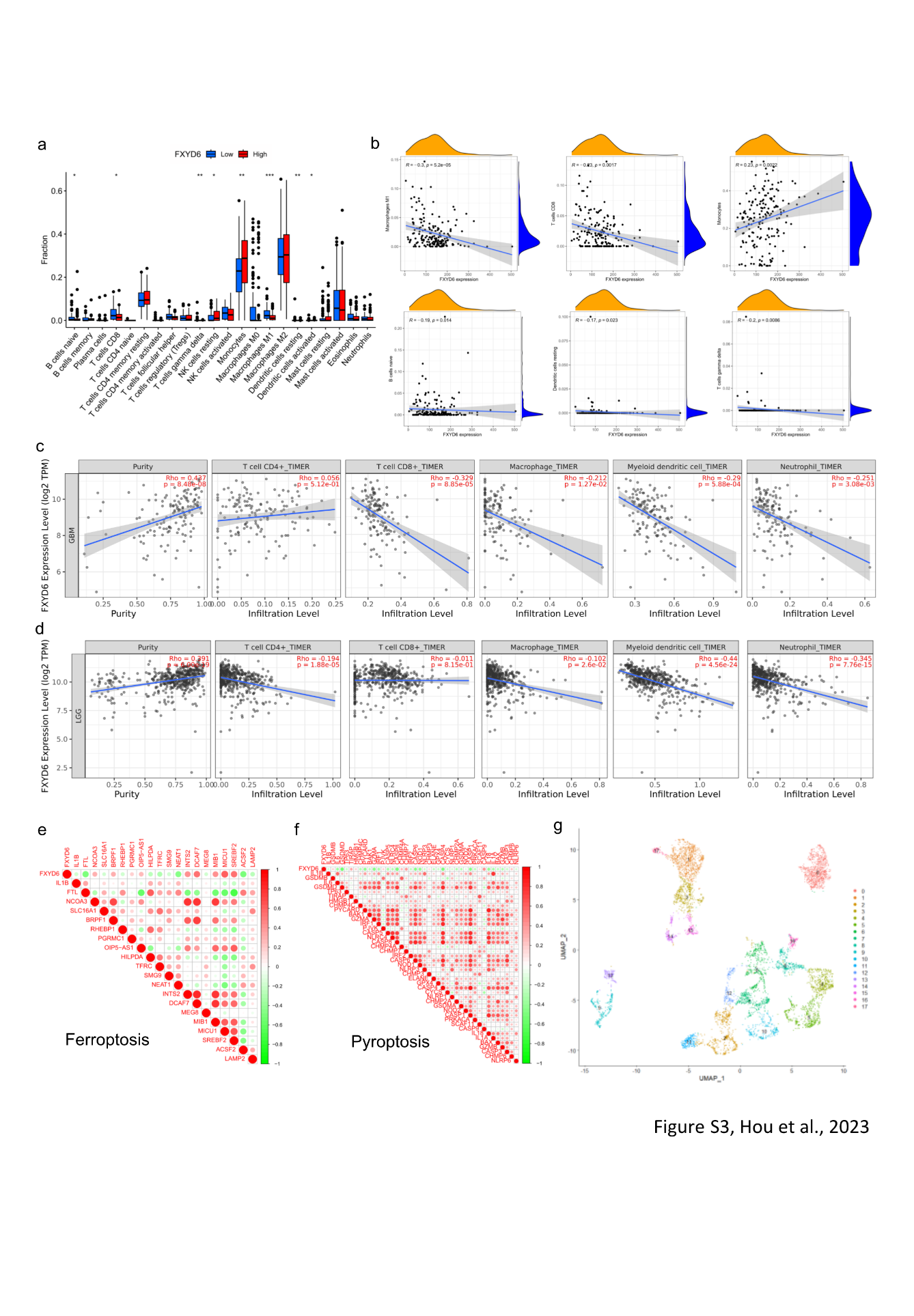

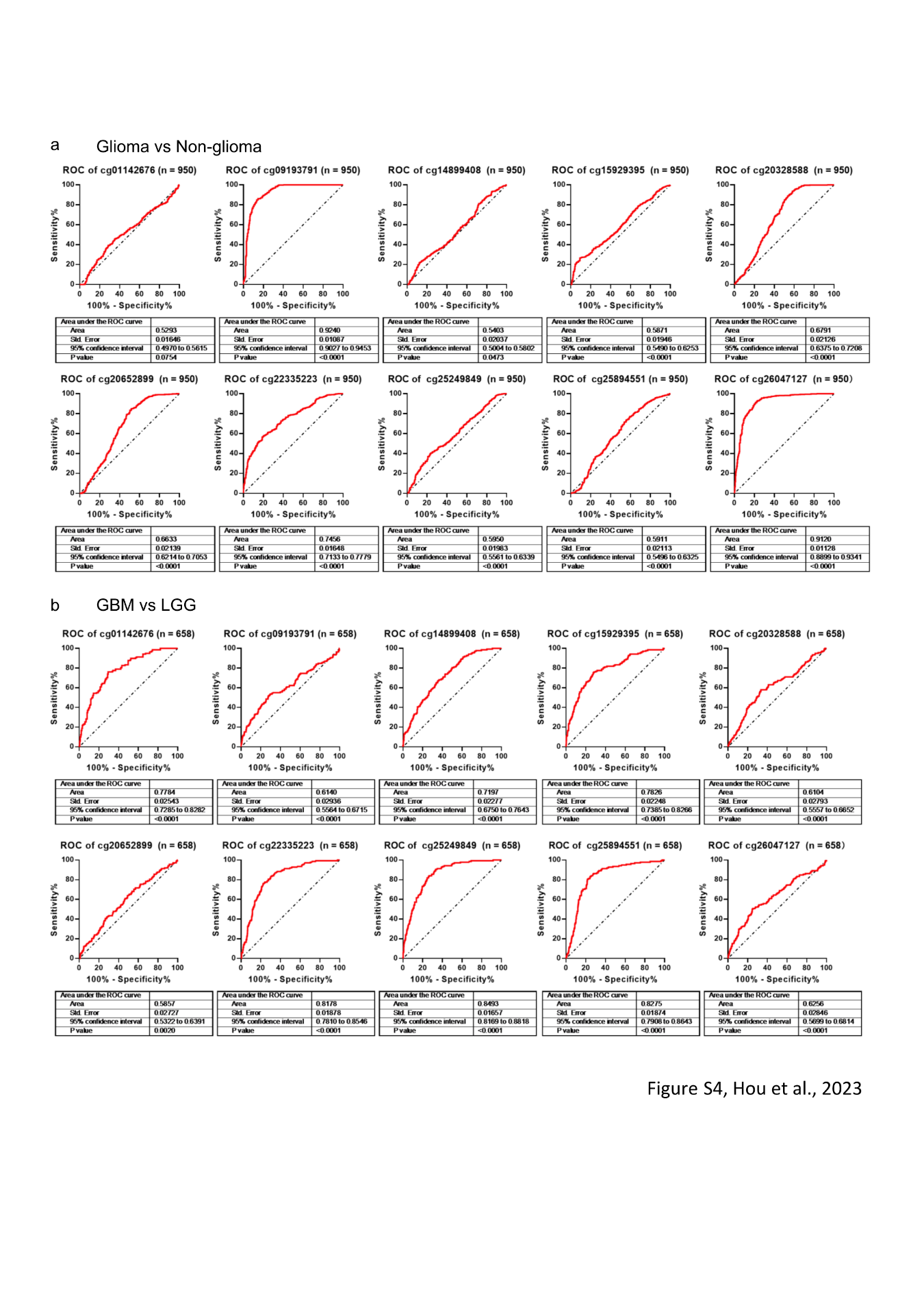

Supplement: Supplementary file 1 — Figure S1. [file CAM4-12-22170-s001.docx]
